# Personal

**Published:** 1911-01

**Authors:** 


### PERSONAL

Dr. L. G. Hanley, of Buffalo, delivered the address to the graduates of Mount Saint Mary's Hospital, Niagara Falls, at the recent commencement exercises held in the auditorium of the Shredded Wheat Building.

---

Dr. Charles Lybrand Bonifield, has been appointed a member of the Board of Medical Directors of the Cincinnati Hospital, in place of Dr. Nathaniel Pendleton Dandridge, deceased.

---

Dr. Clayton K. Haskell, Chief Surgeon of the New York State Soldiers' Home at Bath, has tendered his resignation. The Board of Managers at its meeting in January, 1911, will fill the vacancy thus occasioned.

---

Dr. Edgar A. Vander Veer, of Albany, accompanied by Mrs. Vander Veer, spent two or three days in Buffalo in the early days of December. Dr. Vander Veer read a paper relating to hospital economics at the midyear meeting of the American Academy of Medicine. Dr. and Mrs. Vander Veer visited Niagara Falls before returning to Albany.

---

Dr. Julius Y. Cohen, of Buffalo, has gone abroad, sailing recently on the Kaiserin Auguste Victoria, for a year's study in Vienna.

---

Dr. John L. Heffron, of Syracuse, Dean and Professor of Clinical Medicine in the medical school of Syracuse University, recently visited Buffalo and spent a day or two in attendance upon the meeting of the American Academy of Medicine held here in early December. He read a paper before that body.

